# A Review on Fish Skin-Derived Gelatin: Elucidating the Gelatin Peptides—Preparation, Bioactivity, Mechanistic Insights, and Strategies for Stability Improvement

**DOI:** 10.3390/foods13172793

**Published:** 2024-09-02

**Authors:** Jean Mary Joy, Amruth Padmaprakashan, Akshay Pradeep, Preethy Treesa Paul, Rosemol Jacob Mannuthy, Suseela Mathew

**Affiliations:** 1 Biochemistry and Nutrition Division, ICAR-Central Institute of Fisheries Technology, Cochin 682029, Kerala, India; mailtojeanmary@gmail.com (J.M.J.); amrithprakash.edu@gmail.com (A.P.); akshay.cift@gmail.com (A.P.); preethyambalan@gmail.com (P.T.P.); rosemol529@gmail.com (R.J.M.); 2 Faculty of Marine Sciences, Cochin University of Science and Technology, Cochin 682022, Kerala, India; 3 Department of Zoology, St. Teresa’s College (Autonomous), Ernakulam 682011, Kerala, India; 4 Department of Life Sciences, Christ University, Hosur Main Road, Bhavani Nagar, Bangalore 560029, Karnataka, India

**Keywords:** gelatin, fish skin, characteristics, applications, limitations, gelatin peptides, bio-functionality, stability

## Abstract

Fish skin-derived gelatin has garnered significant attention recently due to its abundant availability and promising bioactive properties. This comprehensive review elucidates various intricacies concerning fish skin-derived gelatin peptides, including their preparation techniques, bioactive profiles, underlying mechanisms, and methods for stability enhancement. The review investigates diverse extraction methods and processing approaches for acquiring gelatin peptides from fish skin, emphasizing their impact on the peptide composition and functional characteristics. Furthermore, the review examines the manifold bioactivities demonstrated by fish skin-derived gelatin peptides, encompassing antioxidant, antimicrobial, anti-inflammatory, and anticancer properties, elucidating their potential roles in functional food products, pharmaceuticals, and nutraceuticals. Further, mechanistic insights into the functioning of gelatin peptides are explored, shedding light on their interactions with biological targets and pathways. Additionally, strategies aimed at improving the stability of gelatin peptides, such as encapsulation, modification, and integration into delivery systems, are discussed to extend the shelf life and preserve the bioactivity. Overall, this comprehensive review offers valuable insights into using fish skin-derived gelatin peptides as functional ingredients, providing perspectives for future research endeavors and industrial applications within food science, health, and biotechnology.

## 1. Introduction

The term “gelatin” refers to a group of protein fractions obtained via partial hydrolysis of collagen, a naturally occurring structural protein primarily present in the connective tissues of animals [1]. Among the many abundant animal proteins, collagen, the precursor of gelatin, stands out as the most prevalent protein and is the most extensively employed biopolymer [2]. Gelatin is employed in various uses, such as serving as a component of food products, producing pharmaceuticals, contributing to advancements in the biomedical sector, and finding applications in a wide array of non-food sectors. This popularity originates from its potential to form gels, emulsions, and stabilizers and modulate the texture of the constituents it is augmented with [3]. Carbohydrates like starch, alginate, pectin, agar, and carrageenan are other gelling agents, where the gels typically have significantly higher melting temperatures and cannot dissolve below body temperature [4]. The distinctive properties of gelatin, such as its ability to melt below the normal physiological temperature, create a unique “melt-in-mouth” sensation that enhances the release of flavor and aroma [5]. This specific characteristic represents one of the fundamental defining attributes of products made with gelatin, including gelatin desserts, and poses challenges in terms of replication by alternative biopolymer materials.

The global gelatin industry continues to expand, nearing a market value of approximately USD 5 billion, with projections indicating an anticipated rise to USD 6.67 billion by the year 2027 [6]. The primary source of gelatin is acquired from the bones and skins of cattle and pigs, with pig skin being the preferred and most commonly used option in the preceding years [7]. The commercial production of gelatin from mammalian sources has demonstrated a detrimental impact as a result of the mad cow disease outbreak and certain religious constraints [8]. Albeit alternative raw materials for gelatin production exist, such as by-products originating from the poultry and fish-processing sectors, these alternative sources offer supplementary avenues for gelatin production alongside traditional sources from pigs and cattle [9,10]. Fish gelatin has reduced allergenicity compared to conventional gelatin, and broader cultural and religious acceptance, with higher bioactivity [11].

## 2. Collagen: The Precursor Molecule to Gelatin

**Figure 1:**
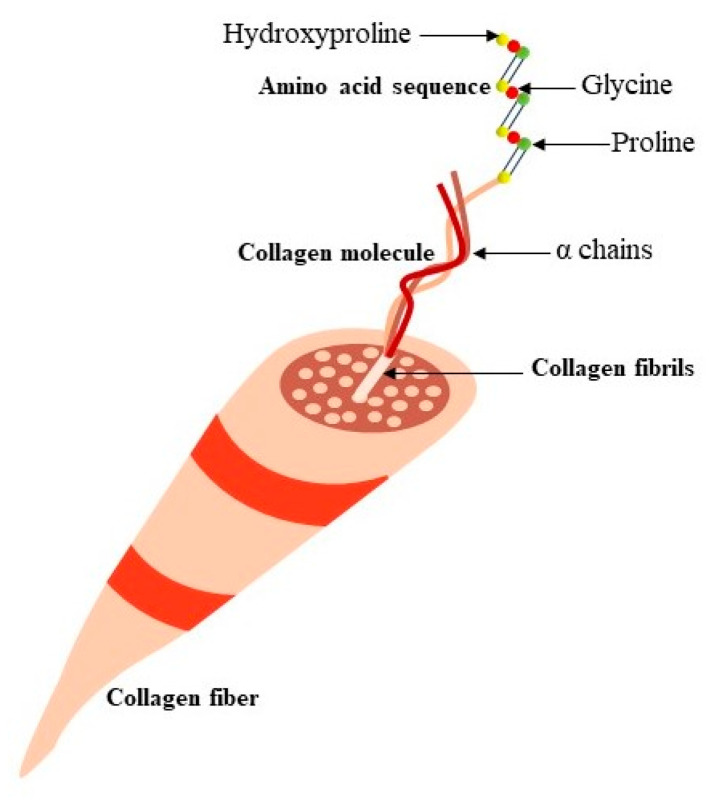
Molecular structural orientation of collagen.

Gelatin and collagen possess remarkable similarities in their chemical composition, as gelatin is extracted from collagen by partial hydrolysis [12,13]. Figure 1 represents the molecular orientation of collagen. Collagen comprises about 20–25% of the overall protein content found in mammals. This protein is notable for its unique amino acid composition, especially due to the presence of two altered amino acids, hydroxyproline and hydroxylysine [14]. These specific amino acids are essential for the stability and functionality of collagen, aiding in its structural integrity and role in providing strength and support to various bodily tissues. The molecular structure of collagen primarily consists of a repetitive sequence denoted as “Glycine-X-Y,” where “X” frequently represents proline and “Y” often denotes hydroxyproline. The primary attribute of collagen resides in its unique triple-helix configuration, constructed by a specialized helical arrangement composed of three polypeptide chains containing a significant proportion of imino acids. Each polypeptide chain adopts a left-handed conformation and consists of three amino acids per turn. These three polypeptide chains, denoted as α-chains, intertwine in a super-twisted manner, forming a right-handed superhelix known as tropocollagen [15], which is the fundamental building block of collagen. Its molecular weight is approximately 330 kDa, with dimensions of roughly 300 nm in length and about 1.5 nm in diameter [16]. 

**Figure 2:**
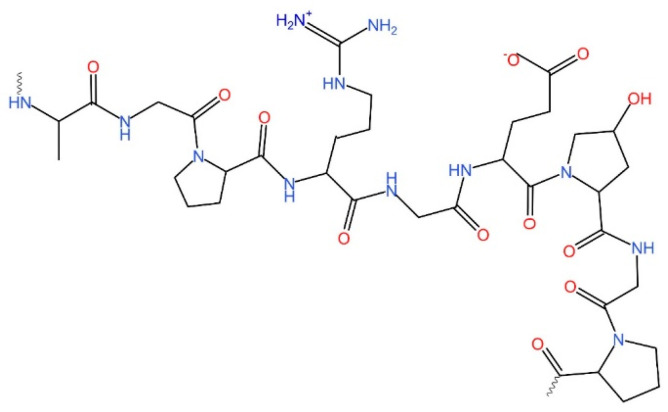
Basic chemical structure of gelatin.

In the process of gelatin production, gelatin is extracted from pretreated collagenous tissue using hot water, causing the collagen’s triple-helical structure to lose its integrity and break down into individual soluble chains and smaller polymers or fragments. Hence, gelatin constitutes a different range of fractions and peptide chains despite its composition being a singular collagen fraction. Upon cooling, these chains can reassemble into fresh triple-helical structures. However, they might not perfectly match the original collagen structure, limiting how well the triple helix can be re-established. This process causes an irreversible disruption to the architecture of collagen fibrils, ultimately leading to the creation of gelatin. The structural breakdown prevents the collagen from returning to its natural state, making it impossible for the triple-helix configuration to reform completely, and as a result, gelatin is produced [17,18]. Figure 2 represents the basic chemical structure of gelatin.

## 3. Collagen-to-Gelatin Extraction Protocol

**Figure 3:**
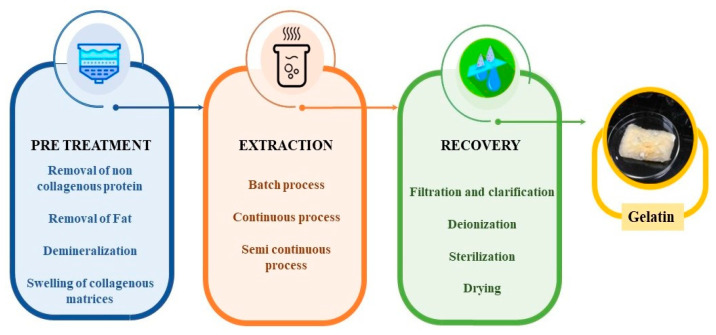
Overview of the gelatin extraction process.

The gelatin production process conventionally involves three distinct processing phases, as delineated in Figure 3. These phases commence with preliminary pretreatment operations to eliminate non-collagenous contaminants and prepare the collagen for subsequent extraction. Subsequently, aqueous extraction techniques are utilized to transform the collagen into gelatin. Following this, a series of purification and extraction processes are carried out to produce a highly refined and thoroughly dried form of gelatin. These steps ensure the final product is impurity-free and meets the desired quality standards for various applications [1]. During pretreatment, the raw materials undergo extensive water washing to eliminate visible impurities. Subsequently, they undergo acid, alkali, neutral salt, or enzyme pretreatments [14,19,20,21,22,23,24,25]. The initial pretreatment steps fulfill two primary functions: first, to alter the collagen structure by breaking down the intramolecular cross-links, such as covalent and hydrogen bonds, and second, to remove additional contaminants. Furthermore, in certain scenarios, size-reduction techniques may be employed to improve the overall efficiency of the process [26]. Following these pretreatments, the second phase involves gelatin extraction using hot water, typically at temperatures above 40 °C. To achieve an improved gelatin yield, the extraction process has been designed to optimize conditions like the temperature, pH, and extraction time [26]. During the final phase, the extracted gelatin undergoes a series of separation techniques, which include filtration, purification, and drying. Filtration eliminates insoluble substances, such as unhydrolyzed collagen and lipids. To effectively eliminate inorganic salts, purification is accomplished using ion exchange and ultrafiltration columns. Subsequently, the gelatin is subjected to drying and grinding processes. 

## 4. Rationale behind the Search for Alternatives to Commercial Gelatin

A group of consumers avoids gelatin-containing products like yoghurt, desserts, spreads, and ice creams due to their origin. The annual global gelatin production approximated 326,000 metric tons, predominantly sourced from pigskin (46%), bovine hides (29.4%), and bones (23.1%) [6]. Gelatin from pigskin is not accepted in Judaism and Islam, while cattle-derived gelatin entails religious compliance [1]. Furthermore, there exist concerns related to bovine spongiform encephalopathy (BSE), also known as mad cow disease, which poses a significant challenge to the development of these industries. Despite these challenges, there is a growing global demand for collagen and gelatin products concerning their exceptional techno-functional attributes.

## 5. Exploring Fish Gelatin as a Feasible Replacement for Mammalian Gelatin

India produces around 2 million metric tons of fish waste [27]. The effective utilization of by-products significantly influences the economy of the country and its environmental pollution levels. Moreover, effective management of post-harvest loss remains an impressive strategy to improve the fishery production of the country, which contributes to the GDP of the nation. Failure to utilize or underutilization of these by-products results in missed revenue opportunities and contributes to the growing costs associated with their disposal. Ongoing research aims to explore the potential applications of various fish-processing by-products. Despite several existing methods, valuable components, with protein being particularly noteworthy, can be extracted on a large scale from fish-processing by-products. Gelatin has gained critical prominence in this regard [28]. 

There are numerous sources of gelatin in both freshwater and marine habitats. Fishes, marine invertebrates, and sea mammals comprise the three main categories of species used to make gelatin [29]. According to their preferred habitats, fish are often divided into warm-water fish and cold-water fish. Cold-water species like pollock, cod, and salmon play a key role in commercial fish harvesting. They are frequently processed to produce skinless, boneless fillets, which generate a considerable amount of fish scales, skin, and bones as waste, which are used to produce gelatin. Warm-water fishes are primarily responsible for the majority of freshwater fish aquaculture production, and in the present context, a significant portion of the commercial fish gelatin supply is sourced from these warm-water fish species [1].

Fish skin, which accounts for about 8–10% of the total fish weight, represents a significant by-product of the fish-filleting industry [30]. Traditionally, fish skin has primarily been used as animal feed. However, fish skin is high in protein, primarily collagen, making it a promising source for gelatin production and adding considerable value to this by-product. Over recent years, substantial research has been conducted regarding the extraction of gelatin from fish skin, and the outputs have been promising [13,20,31,32,33,34,35]. Gelatin production has been achieved using skin from a range of warm-water fish species, including catfish, Nile perch, shark, tuna, megrim, and tilapia, as well as cold-water fish such as hake, cod, Alaska pollock, and salmon [3]. Extensive research on gelatin extraction has provided valuable insights into the potential skin sources from various fish species, with the extraction yields ranging from 2 to 20% via various extraction methods, as depicted in Table 1.

## 6. Characteristics of Fish Gelatin 

### 6.1. Physical Attributes

a. Melting and gelling temperature: Gelatin creates and maintains stable three-dimensional gels through hydrogen bonds with water [51]. The temperatures at which gelatin melts and gels are directly related to the levels of proline and hydroxyproline present in the initial collagen [11]. These specific amino acids significantly influence the thermal properties of gelatin, determining how it behaves under different temperature conditions. Higher concentrations of proline and hydroxyproline typically result in gelatin with higher melting and gelling points, reflecting the stability and structural integrity derived from the original collagen source. The key difference between mammal and fish gelatins lies in their gelation temperatures. Mammal gelatin has about 30%, warm-water fish gelatin has 22–25%, and cold-water fish gelatin has about 17% proline and hydroxyproline. Cold-water fish gelatin has lower melting and gelling points due to the lower proline and hydroxyproline content, as in the case of cod fish, which reduces the propensity for intermolecular helix formation. Warm-water fish gelatin like that from tilapia and Nile perch behaves like mammalian gelatin due to the comparable proline and hydroxyproline content. For instance, Nile perch (*Lates niloticus*) skin has the potential to serve as an excellent source of gelatin due to its rich imino acid content, which imparts strong gelling properties. Research by Norziah et al. [52] found cod skin gelatin had lower melting points than bovine and shortfin scad gelatins. The ability of gelatin to melt below body temperature is advantageous for the food industry, which improves the flavor release during consumption and influences the sensory experience in food products. The study by Choi and Regenstein [53] demonstrated that the decreased melting point of cold-water fish gelatin contributes to an improved flavor release, enhanced fruit aroma, and faster melt rate in water-based gel desserts.
b. Gel strength: An important physical property of gelatin is its gel strength, which is affected by several factors, including the molecular weight and complex interactions determined by the amino acid composition and the ratio of α/β-chains within the gelatin matrix [54]. Schrieber and Gareis [55] detailed that gel strength largely depends on the concentration of components with molecular weights around 100,000 g/mol. Moreover, gelatin with a higher content of α-chains generally exhibits greater gel strength. In contrast, an increased presence of peptides with molecular weights either significantly above or below those of α-chains tends to reduce the gel strength [56]. The gelation strength of commercial gelatins is quantified through the bloom values. The higher the bloom value, the stronger the gel. The bloom value can be categorized into three groups: high (220 to 300 bloom), medium (150 to 220 bloom), or low (<150 bloom). Furthermore, gelatin possessing a higher viscosity is deemed more commercially desirable and, as a result, commands a premium price in the market [57]. Yellowfin tuna skin is considered a suitable raw material for producing gelatin with high gel strength [58]. Table 2 illustrates the diverse functional attributes of gelatin-based food products as determined by their gel strength.

The gelatin production process conventionally involves three distinct processing phases, as delineated in Figure 3. These phases commence with preliminary pretreatment operations.

c. Viscosity: Viscosity is a crucial functional characteristic for process control. A gelatin solution’s ability to gel depends on the temperature and the viscosity of the gelatin in the water [60]. The species of fish, habitat, origin, and amino acid content are a few of the variables that might affect whether fish gelatin has gelling or non-gelling qualities [3]. The viscosity of gelatin derived from cold-water fish is very high, which may not be appropriate for numerous applications [61]. In contrast to the gel strength, the viscosity demonstrates a weaker correlation with textural characteristics. The molecular weight distribution predominantly influences the viscosity. Gelatin samples characterized by substantial molecular weight fractions yield elevated viscosity; however, this does not inevitably translate to equally high gel strength [61].
d. pH: The pH is an important physical parameter that influences the quality of gelatin. The production process influences the pH of gelatin, and this pH range determines the types of gelatins:
  (i) Type A: It is obtained through the acid processing of collagenous raw materials, featuring pH values ranging from 3.8 to 5.5 [62].
  (ii) Type B: Produced by alkaline processing of collagenous raw materials, and it possesses pH values ranging from 5 to 7.5 [62].

It is worth emphasizing that combinations of types A and B, as well as gelatins resulting from modifications of the mentioned processes, may diverge from the specified pH ranges [63].

e. Thermoreversibility: Gelatin falls into the group of physical gels involving Van der Waals forces and hydrogen bonds; the interactions or bonds between the constituent chains are primarily of a physical nature, with an energy of approximately 2 Kcal/mol. The bonding energy within gelatin is relatively modest, which enables gelatin to form thermoreversible gels. When compared to other gel-forming substances like proteins and polysaccharides, gelatin has greater thermoreversibility due to the non-random presence of imino acids (such as proline or hydroxyproline) [64,65,66]. This implies that it achieves softness, transforms into a liquid state, and also reverses to a gel-like consistency upon cooling.
f. Texture: Gelatin is vitreous in gel form, with a taste and smell that are nearly imperceptible. Its dried appearance ranges from pale yellow to white. It is offered in a variety of physical configurations, including sheets, fine powders, and coarse granules [1].
g. Stability: Gelatin remains stable for at least five years when stored [67] properly in sealed containers under typical storage conditions to prevent moisture changes. The bloom value can vary slightly due to modest changes in the moisture levels. These bloom variations, nevertheless, may be precisely computed from the moisture content and do not signify a change in the underlying intensity or quality of the bloom [68].

A comparative analysis of the attributes of gelatin derived from various sources, including warm-water and cold-water fish, porcine, bovine, and poultry varieties, is presented in Table 3.

### 6.2. Chemical Attributes

- Amino acid composition: The amino acid content significantly affects the melting and setting temperatures of gelatin derived from various sources. Proline and hydroxyproline exert notable impacts on both the gel strength and the temperature at which gelling occurs [67]. Furthermore, the levels of aspartic acid, glutamic acid, arginine, histidine, lysine, and hydroxylysine are crucial for the formation of cross-links and electrostatic interactions [77].
- Peptide size and quality: Collagen tissues are treated with acid/alkali, heat, and water to disrupt the fibrils, yielding gelatin’s irreversibly. Gelatin comprises collagen fractions above 30 kDa; lower-molecular-weight forms are gelatin hydrolysates, aiding in gel formation. Heat at ~40 °C frees the α-chains via bond breakage in new collagen while the mature bonds of collagen endure. Higher heat breaks the covalent bonds, yielding smaller α-chains and varied weights. Diverse collagen amino acids cause random bond breaks, causing variability. Raw materials add impurities like proteins, lipids, and minerals; the water in commercial gelatin (9–14%) varies based on the production and storage.

## 7. Versatile Uses and Applications of Gelatin 

### 7.1. Food Industry

The confectionery industry harnesses the versatility of gelatin for various purposes beyond its thermoreversible gelling capabilities. Gelatin is essential to forming and stabilizing foam, as well as binding, emulsifying, and regulating sugar crystallization, in confectionery. The thermoreversible gelling property is especially valuable in confectionery for creating gummy candies, marshmallows, and other gel-based treats. Altan et al. [78] tested rainbow trout skin gelatin (*Oncorhynchus mykiss*) with varying confectionery solute concentrations as an alternative to mammalian gelatin. Adding sorbitol and sucrose (10 g/100 mL) enhanced the rheological characteristics. The gel strength increased from 828 Pa to 2317 Pa and 1996 Pa with sorbitol and sucrose addition. The melting points of sorbitol and sucrose increased from 17.5 °C to 22 °C and 22.1 °C, respectively. Their results suggest that sorbitol and sucrose induced more junction zones, resulting in increased hardness and chewiness, showing potential for using fish gelatin in gummy candies [78]. 

The foam formation and stabilization properties of gelatin make it an essential ingredient in producing aerated confections like marshmallows and nougat. It helps to create and stabilize foamy structures, contributing to the light and fluffy texture of these sweets. In the study by Eddy and Editya [79], gelatin extracted from the skin of *Epihinephelus* sp. was utilized to produce marshmallows. The research findings revealed that marshmallows exhibited specific physical attributes, including a chewiness of 53.62 N, an elasticity of 72.93%, and a density of 0.93 g/mL. Moreover, they possessed organoleptic qualities rated at 3.47 for texture, 3.43 for taste, 3.27 for aroma, and 3.50 for color. In additions, Tinratat and Sila-asna [80] studied fish skin gelatin and found it effective for application in panna cotta products.

In addition, gelatin acts as a binding agent that helps hold together various ingredients in confectionery products. Specifically, to bind nuts, fruits, and other inclusions in bars, fruit gels, and nougats. Gelatin can also improve the emulsification of fat and water-based ingredients in confections. This is beneficial in applications like chocolate and toffee, where it aids in achieving a smooth and creamy texture [81]. 

Moreover, preventing unwanted sugar crystallization is crucial for achieving the desired texture and appearance in the confectionery industry. Gelatin serves as an effective anti-crystallization agent, ensuring that confections like fudges and caramels remain smooth and free of unwanted sugar crystals [82].

Choobkar et al. [83] explored the effectiveness of *Cyprinus carpio* skin gelatin as a substitute for bovine gelatin in pastille manufacturing as it augments the nutritional value. The results showed that 75% carp skin gelatin had a more significant effect on the protein and essential elements, especially phosphorus and iron, compared to 100% commercial bovine gelatin. According to a study by Choi and Regenstein [53], fish gelatin has similar physical and chemical properties to pig gelatin, and it scored higher in a blind sensory test. Additionally, according to their studies, cold-water fish gelatin’s lower melting point improves the fruit scent, flavor release, and melt rate in water gel desserts [53]. By leveraging these diverse properties of gelatin, the confectionery industry can produce a wide range of delightful and consistent treats, each with its own unique texture and taste.

In addition to its role in desserts and jellies, gelatin is critical in enhancing the quality of certain dairy products and pastries, ensuring that these long-lasting items meet the expectations of consumers. Fundamentally, milk is an intricate emulsion of water and oil. Gelatin is incorporated into dairy products to enhance their emulsifying properties by adhering gelatin molecules to the surface of fat droplets, thereby decreasing the surface tension within the aqueous phase [84]. The incorporation of gelatin into dairy items serves multiple purposes. It aids in the retention of whey within the product matrix, preventing the undesired separation of aqueous whey, which is particularly noticeable in yoghurts, curds, and cream cheese. Furthermore, when gelatin is introduced into milk-based desserts, it plays a pivotal role in altering the surface tension of water [85]. This modification facilitates the formation and persistence of foam through mechanical agitation or gas infusion. Gelatin also contributes to the production of ice cream by modifying the dimensions and dispersion of ice crystals formed during freezing, thereby exerting a notable influence on the ultimate mouthfeel and texture of the ice cream product [85].

In yoghurt, tilapia skin gelatin (TSG) was used as an alternative to mammalian gelatin. When compared to bovine gelatin, TSG has lower melting and gelling temperatures. Moreover, tilapia skin gelatin proved highly effective in enhancing the water-holding capacity of acid milk gels. The study concluded that the rheological, water-holding, texturing, and sensory aspects of TSG are comparable and substitutable to bovine gelatin in yoghurt [86]. A similar study by Cheng et al. [87] looked into a low-fat spread made of pectin and fish gelatin. Temperature sweep, bulk density measurements, texture analysis, and morphological assessment were used to study the impacts of salt addition and pH change on this spread. According to the study, lowering the fish gelatin-to-pectin ratio increased the spread’s bulk density, firmness, compressibility, adhesiveness, elasticity, and meltability. Additionally, syneresis occurred in spreads made with high acidity, although it could be prevented by adding sodium chloride.

The inferior melting property of fish gelatin can be employed in various dry products, including microencapsulation [88]. The microencapsulation of vitamins and other pharmaceutical additions like azoxanthine is one of the main uses of fish gelatin. It can also serve as a microencapsulation agent for colorants [57]. Warm-water fish gelatin (150–300 bloom) was used by Soper [89] to microencapsulate food flavors such as vegetable oil, garlic flavor, lemon oil, apple flavor, or black pepper. The production of microencapsulated capsules results from this intricate coacervation process, which occurs at temperatures between 33 and 35 °C. The majority of encapsulation experts have acquired the necessary proficiency to manage fish gelatin within their advanced processes, with relatively small quantities of fish gelatin being employed to produce soft gel capsules. Fish gelatin-based soft capsules are primarily utilized in the realm of dietary supplements [57].

### 7.2. Pharmaceutical and Medical Industry

Gelatin has diverse applications in the field of pharmaceuticals and medical products. It is a key component of plasma substitutes employed in critical care and surgical procedures [90,91]. The perioperative allergic response associated with mammalian gelatin [92] can be eradicated by substituting fish gelatin. Additionally, gelatin is utilized in the manufacturing of vitamin-coated products, pastilles, and tablets, as well as the production of globules, paste dressings, and sponges. Furthermore, it plays an essential role in developing innovative vaccine formulations. Remarkably, approximately 90% of pharmaceutical-grade gelatin is dedicated to the production of capsules, encompassing both soft and hard gelatin capsules [93,94]. In addition, fish gelatin holds promise as a potential ingredient in wound healing and tissue regeneration processes due to its high biodegradability, biocompatibility, and low antigenicity [95]. The gelatin from fish has demonstrated significant prospects in treating various ailments. One such condition is osteoporosis, characterized by bone calcium loss, resulting in increased porosity and fragility, ultimately reducing bone density. Shark skin gelatin has been found to promote bone formation and enhance type 1 collagen, as demonstrated in a study by Nomura et al. [96]. Another investigation by Naomi et al. [95] utilized fish gelatin to mitigate bone brittleness, revealing its substantial potential. Investigations by Kearney et al. [97] and Cheng et al. [87] looked into the possibility that hypertension is a substantial risk factor for cardiovascular diseases, and fish gelatin has the potential to be used as an effective antihypertensive drug for those who suffer from the condition.

### 7.3. Nutritional and Health Benefits

Gelatin is a valuable protein source, devoid of cholesterol and sugar, and virtually fat-free. It is highly digestible and undergoes complete breakdown within the human body. According to studies, gelatin has been employed in food products to increase the protein levels and decrease the carbohydrates, salt, and fat in low-fat foods, while also acting as a vitamin carrier. Gelatin may benefit the locomotion and skeletal systems, notably regarding bones, tendons, cartilage, and ligaments. Furthermore, there is a suggestion that regular consumption of gelatin may contribute to stronger hair and connective tissue, resulting in firm skin, glossy hair, and resilient fingernails [98].

## 8. Challenges Associated with Fish-Derived Gelatin 

Although fish gelatin shows promise as a potential substitute for mammalian gelatin, its widespread commercial adoption has been constrained. Several hurdles must be surmounted before fish gelatin can be widely embraced as a feasible alternative to mammalian gelatin in the food industry [3]. Compared to mammalian gelatin, fish gelatin sourced from cold-water species, which constitute the predominant portion of industrial fisheries, frequently exhibits sub-optimal physical and functional characteristics. These shortcomings include diminished gel strength and lower gelling and melting temperatures. At ambient temperature, gelatin sourced from cold-water fish lacks gelation characteristics. Typically, the gelation of cold-water fish gelatin occurs below 8 to 10 °C, with the exact temperature being contingent upon factors such as th gelatin concentration, average molecular weight, ionic strength, pH, cooling rate, and measurement methodology [99,100]. In contrast to mammalian gelatin, type A fish gelatin, obtained from the skins of cold-water fish species such as cod, haddock, and pollock, exhibits notably lower storage modulus (G0) values, along with reduced gelling temperatures (4–5 °C) and melting temperatures (12–13 °C). These characteristics render such gelatins unsuitable for replacing mammalian gelatin due to the aforementioned attributes [99].

Addressing the challenges of the inadequate gelling properties and notable quality variations among distinct fish species is imperative. As posited by Gómez-Guillén et al. [101], two pivotal considerations arise when utilizing fish skin for gelatin production. Firstly, the collagen molecules within the skin of different fish species exhibit inherent diversity owing to species-specific characteristics. Secondly, in contrast to the more robust collagen present in mammalian sources, collagen derived from fish skin is predisposed to breakdown under chemical treatments (acidic or alkaline) due to the lower levels of intra- and interchain non-reducible cross-links [102,103,104].

## 9. Enhancing Fish Gelatin: Solutions for Its Limitations 

In response to the challenges due to the inferior characteristics of fish gelatin in comparison to commercial mammalian gelatins, three distinct strategies have been put forward:

- Utilizing enzymatic cross-linking methods, such as the application of enzymes like transglutaminase [105], or employing chemical cross-linking approaches with compounds like genipin [61].
- Developing composite gelation systems involves blending fish gelatin with high-bloom gelatins, as substantiated by the research conducted by P. Gilsenan [106], P. M. Gilsenan and Ross-Murphy [107] and Zhou et al. [108]. Alternatively, these systems can incorporate appropriate plant hydrocolloids, as exemplified by Haug et al. [109] and Pranoto et al. [110]. These methods promise to augment the gel strength, improve the gelling properties, and elevate the melting temperature.
- Modifying the properties of gelatin through the introduction of solutes, such as various types of salts, as deliberated by Elysée-Collen and Lencki [111].

Table 4 displays the methodologies employed to alter the techno-functional characteristics of gelatin derived from fish skin, aiming to customize its properties.

## 10. Bioactive Peptides Derived from Gelatin: Their Potential Efficacies 

Dietary proteins harbor biologically active peptides that are latent within the native protein structure but can be liberated through gastrointestinal enzymatic hydrolysis, food processing, or microbial fermentation. Upon liberation, these bioactive peptides can modulate diverse physiological processes within the organism. Gelatin has attracted considerable interest as a reservoir of such bioactive peptides, presenting promising health advantages suitable for integration into both nutritional and pharmaceutical contexts [5].

One widely used approach for obtaining bioactive peptides involves enzymatic protein digestion, carried out under carefully controlled time and temperature conditions using specific enzymes [122]. Typically, bioactive peptides are dormant within their parent proteins, but they become active when they are 2–20 amino acids in length [123]. The bioactivity of these peptides is influenced by their amino acid composition, sequence, and molecular mass and can also be impacted by the processing conditions [124].

## 11. Production Methods for Fish Gelatin Hydrolysates and Peptides

**Figure 4:**
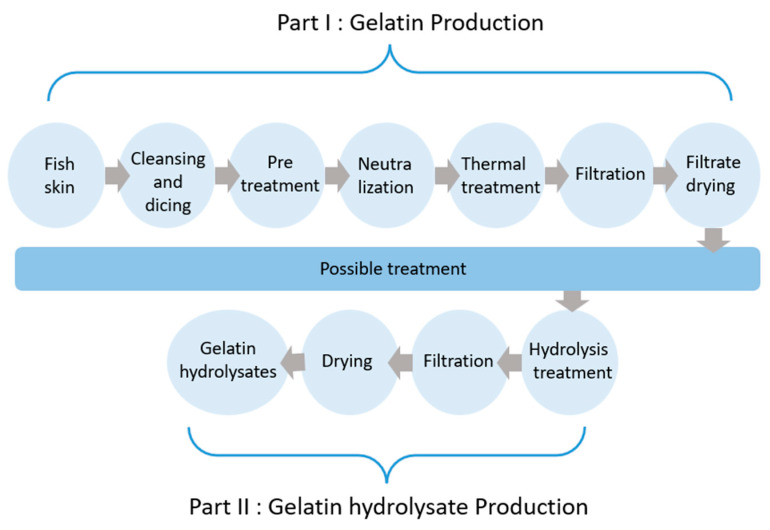
Gelatin hydrolysate production.

The effective hydrolyzation of native collagen with an intact triple-helical structure requires pretreatment to disrupt the molecular order viz the secondary structure of the protein to promote its degradation. For food and pharmaceutical applications, physical pre-processing technologies that are benign to the environment are best. Physical pretreatments promote gelatin hydrolysis, resulting in higher hydrolysis rates and improved functional and nutraceutical properties in the hydrolysates that are produced [125]. Figure 4 illustrates the procedural stages integral to the production process of gelatin hydrolysate. Various methodologies for physical treatment are examined and discussed herein.

a. Ultrasonication

Ultrasonication is a prominent method for accelerating enzymatic reactions by modulating the concentration proportions of enzymes and substrates. Employing sonication as a preliminary step for the enzymatic fish gelatin hydrolysis can provide functional properties such as enhanced hydrolysis, recovery, and bioactivity [126,127]. Investigation into the impacts of ultrasonic pretreatment on the production of fish gelatin hydrolysates elucidated that this procedure escalates the reaction rate constant while reducing the activation energy requisite for hydrolysis, concomitant with an elevation in the enzyme deactivation energy. Furthermore, ultrasonic pretreatment elicits alterations in the α-helix and β-turn structures through physical and chemical mechanisms [128,129].

b. Microwave pretreatment

Microwave pretreatment is a physical pretreatment strategy to hasten gelatin hydrolysis and increase its degree of hydrolysis. The extent of the native structure disruption is influenced by the frequency, power, and exposure time. The enzyme-binding sites in the gelatin structure are revealed, and the hydrolysis rate is accelerated, which decreases the hydrolysis time and boosts the yield [130].

c. Hydrothermal pretreatment

Hydrothermal pretreatment represents a methodology aimed at augmenting the yield and potentially enhancing the functional attributes of fish gelatin hydrolysates (FGHs). Due to this treatment, water molecules undergo a change in the hydrogen bond structure, which increases the presence of ionic products (H3O+ and OH−), allowing them to function as acid or basic catalysts [131,132,133]. The autoclave batch reactor is an efficient instrument for hydrothermal processing that does away with the necessity for a pumping system. However, the heating and cooling times for this device are prolonged. Supercritical water, an environmentally friendly method that uses water as a solvent, provides an additional choice for hydrothermal pretreatment. This method is quick, affordable, and very successful. Elevated pressure and temperature conditions within supercritical water induce the unfolding of gelatin molecules and their subsequent hydrolysis into peptides. This methodology has been employed in a single study to enhance the hydrolysis of fish gelatin [134].

d. High pressure (HP)

The HP technique possesses the capability to induce structural destabilization within the triple-helix arrangement by unfolding the α-chains, thereby exposing concealed molecular sites susceptible to enzymatic action [135]. It has been empirically evidenced that, alongside the acknowledged advantages of the HP approach in other contexts, it can mitigate the antigenic potential of the resultant bioactive peptides (BPs) [136,137]. However, the specific impact of this process on fish gelatin hydrolysates (FGHs) has not been extensively explored within the realm of scientific inquiry until now. To the best of our knowledge, only one investigation [138] has addressed the influence of HP pretreatment on the enzymatic hydrolysis of fish gelatin. The findings of this study indicate that HP treatment enhances both the antioxidant properties and the extent of hydrolysis of FGHs, with the degree of hydrolysis ranging between 5% and 10% across different enzyme systems.

Diverse processes are employed to produce fish gelatin hydrolysates (FPHs), each of which helps break down fish protein into more digestible and functional forms. These techniques encompass autolysis, thermal hydrolysis, bacterial fermentation, and enzymatic hydrolysis.

e. Autolysis

Fish proteins naturally degrade through a process known as autolysis, which is triggered by endogenous enzymes that are found in fish tissue. This can happen after the fish is caught but before there is severe spoiling. The properties of FPHs are influenced by the release of different bioactive substances and peptides during autolysis [139].

f. Thermal Hydrolysis

By exposing fish proteins to high temperatures, a process known as thermal hydrolysis, they may partially break down. The heat causes the protein’s structure to be disturbed, which results in the creation of peptides with different functions. Additionally, this technique can make it easier to extract proteins and bioactive substances [140].

g. Bacterial Fermentation

In bacterial fermentation, particular bacterial strains are used to break down the proteins in fish through their metabolic processes. During the course of their metabolism and growth, bacteria release enzymes that break down proteins into smaller parts. The flavor, aroma, and nutritional content of the finished FPHs can all be improved by this treatment [141].

h. Enzymatic hydrolysis

The peptide linkages in fish proteins are specifically targeted and broken down by the proteolytic enzymes used in this technique. Proteases like trypsin, pepsin, and Alcalase enzymatically break down collagen and gelatin into active peptides, breaking the proteins into smaller peptides and amino acids. With this strategy, the hydrolysis procedure may be precisely controlled, resulting in the synthesis of FPHs with the appropriate characteristics [142].

i. Subcritical water hydrolysis

Subcritical water hydrolysis (SWH) offers an eco-friendlier alternative to traditional hydrolysis methods. This technique can release bioactive peptides from various protein sources, including fish by-products. Subcritical water, also referred to as hot-compressed or pressurized hot water, is water maintained above its boiling point at 1 atm (>100 °C at 0.1 MPa) but below its critical point (374 °C at 22 MPa), with sufficient pressure to keep it in a liquid state. Under these conditions, the water’s dielectric constant decreases with the rising temperature, allowing it to dissolve moderately polar to non-polar substances. Moreover, the ionic product of water increases significantly, enhancing its ability to function as both an acid and base catalyst, which facilitates protein hydrolysis [143].

## 12. Fish Gelatin Hydrolysates and Its Significance for Food and Biomedical Applications

Each year, more than 50% of the biomass produced from fish processing is discarded as waste, showing a significant environmental challenge in the context of the immense market for fish production [144,145,146]. However, these by-products hold great potential for conversion into valuable substances, including amino acids, proteins, and bioactive peptides. Extensive research has already explored the functional properties of bioactive peptides obtained from fish skin gelatin hydrolysate, which have significant applications in the pharmaceutical and food sectors. To exist, fish skin gelatin bioactive peptides find application prospects in various aspects of the food industry, such as food packaging and processing. Additionally, bioactive peptides derived from fish gelatin have demonstrated promise in numerous therapeutic uses, including antioxidative, anticancer, antihypertensive, hypoglycemic, and immunomodulatory properties and numerous others [125,147,148,149]. These bioactive peptides retain the essential structural elements of their parent proteins. Enzymatic hydrolysis to extract active peptides can be performed during food processing by employing either the body’s natural gastrointestinal digestive proteases or external proteases.

The bioactive peptides obtained from enzymatically degraded gelatin from fish skin exhibit considerable promise for applications in developing functional foods, nutraceuticals, cosmeceuticals, and therapeutic agents intended for human utilization. This promise stems from their inherent biocompatibility, heightened bioavailability, safety profile, and targeted efficacy across diverse biological targets [125,150].

## 13. Bio-Functionalities of Peptides Derived from Distinct Fish Skin Gelatin Sources 

The food and pharmaceutical industries have benefited greatly from the extensive research on the possible uses of bioactive peptides isolated from fish gelatin hydrolysate (FGH). These peptides have tremendous potential in numerous areas of the food industry, including food processing and packaging. They also have proven therapeutic properties, such as antioxidative, antihypertensive, hypoglycemic, and several other functions (Table 5). Their high bioavailability, safety, specific actions on many targets, and biocompatibility further increase their potential for usage as therapeutic agents, nutraceuticals, cosmeceuticals, and functional foods for human consumption.

a. Antioxidant

Enzymatically hydrolyzed fish gelatin peptides show exemplary antioxidant potential in various radical systems [173]. To date, the identified antioxidant peptides are in the molecular range of 0.5 to 1.5 KDa, with a 5–16 amino acid sequence. The elucidated antioxidant peptides contain Tyr, Pro, or His in the sequence, and the N-terminus consists of hydrophobic amino acids, mostly Val and Leu. Tyrosine has a substantial role in the antioxidant defense mechanism via radical chain termination by donating electrons from its phenolic lateral chains [174]. Numerous research studies have portrayed the antioxidant potential of fish derived peptides. Table 5a lists the fish sources and enzymes for producing peptides and the sequenced peptides. The antioxidant potentiality was confirmatory detected by more than one oxidative system, such as 1,1-diphenyl-2-picryl hydrazyl (DPPH) free radical scavenging, ferric-reducing antioxidant power (FRAP), hydroxyl radical scavenging, superoxide radical scavenging, metal-chelating activity, thiobarbituric acid reactive substance (TBARS) prevention, linoleic acid peroxidation inhibition, 2,2-azino-bis(3-ethylbenzthiazoline-6-sulfonic acid) (ABTS) radical scavenging, reactive oxygen species (ROS) scavenging, peroxyl radical scavenging, lipid peroxidation inhibition activity and linoleic acid auto-oxidation inhibition.

b. Antihypertensive

Various studies have investigated the potentiality of fish peptides/hydrolysates to inhibit the activity of angiotensin-converting enzyme I. The dipeptidyl carboxy peptidase angiotensin-converting enzyme I transforms the weak vasoconstrictor angiotensin I into the potent decapeptide angiotensin II. It also increases the blood pressure by inhibiting bradykinin, a peptide that lowers the blood pressure. Artificial angiotensin-converting enzyme (ACE) inhibitors are employed in hypertension treatment, yet they are associated with a higher incidence of side effects. Fish-hydrolyzed peptides with antihypertensive activity are a natural substitute for synthetic ones. Many studies have investigated the impact of hydrolysates from fish skin gelatin on blood pressure regulation and their potential as antihypertensive agents (Table 5b).

c. Antimicrobial

Enzymatically derived antimicrobial peptides from fish hydrolysates have a molecular weight mostly below 10 KDa and possess < 50 amino acids in their sequence. Though fish belong to aquatic habitats that are exposed to diverse microbial communities, the innate immune system, with factors like antimicrobial peptides, in fish plays a significant role in the host defense mechanism by killing the microbes on exposure to pathogens. Defensin, piscidin, parasin, hepcidin, and cathelicidin are the major AMPS families. The antimicrobial activity of the peptides is commonly screened by agar diffusion assay by measuring the zone of inhibition. Numerous studies elucidated the bacteriostatic potentiality of fish AMPs against Gram-positive and Gram-negative microorganisms, but few have been conducted on fish gelatin hydrolysates/peptides (Table 5c).

d. Antidiabetic

Diabetes is currently recognized as a serious metabolic condition. Although there are various pharmaceutical treatments available for this illness, they frequently have unavoidable adverse effects. Due to their inherent composition, bioactive peptides obtained from dietary sources have demonstrated significantly fewer adverse impacts. These bioactive peptides have been shown to have the ability to effectively manage diabetes by inhibiting digestive enzymes, lowering insulin absorption, and blocking dipeptidyl peptidase IV (DPP-IV) activity, among other antidiabetic processes (Table 5d). The fish habitat plays a crucial role in influencing gelatin and its bioactive peptides, ultimately affecting the DPP-IV inhibitory activity of fish skin gelatin hydrolysate. Within this sector, researchers and manufacturers may seek to investigate the variances between hydrolysates/peptides derived from cold-water and warm-water fish skin gelatin, as consumer demand emphasizes uniformity in the end product. Warm-water fish skin gelatin, characterized by its rich amino acid content, presents as a more potent reservoir of DPP-IV inhibitory peptides when compared to its cold-water counterpart.

## 14. Stability Improvement Strategies for Peptides

**Figure 5:**
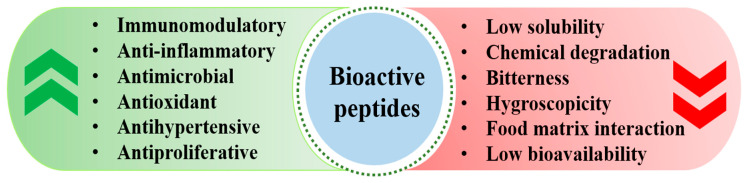
Factors and impediments that restrict the utilization of bioactive peptides in nutraceutical and food applications. Note: Green: benefits of bioactive peptides, Red: limitations of bioactive peptides.

In addition to the immunomodulatory, antioxidant, antihypertensive, anti-inflammatory, antimicrobial, and antiproliferative activities exhibited by bioactive peptides, their utilization of peptides in nutraceuticals and commercial products is significantly impacted by several intrinsic and extrinsic factors (Figure 5). One primary challenge is their susceptibility to chemical degradation, which can occur due to variations in the pH and enzymatic activity. Peptides are often sensitive to changes in the pH, with extreme acidic or alkaline conditions leading to hydrolysis and subsequent loss of bioactivity. Enzymatic degradation from endogenous digestive enzymes and during food processing further compounds this issue by breaking down peptides into less active or inactive forms. This degradation diminishes the peptides’ efficacy and reduces their potential benefits in nutraceutical applications. Interactions with the food matrix also pose considerable challenges. Peptides can interact with various components within the food matrix, such as proteins, lipids, and carbohydrates. These interactions may result in peptide aggregation, alteration of their structure, or reduced solubility, thereby impacting their stability and functionality. For instance, peptides that bind to other macromolecules can become less bioavailable, diminishing their therapeutic effects.

Limited water solubility is another significant constraint. Many peptides exhibit poor solubility in aqueous environments, affecting their incorporation into liquid formulations and limiting their effectiveness in such products. This low solubility can lead to incomplete dissolution and uneven distribution within the product, thereby impacting both the sensory quality and the bioavailability of the peptides. Additionally, the hygroscopic nature of some peptides can adversely affect product quality. Peptides that absorb moisture from the environment can cause texture and consistency changes, leading to clumping and reduced shelf life. This hygroscopic behavior necessitates using specific packaging and formulation strategies to maintain the product stability and performance. Moreover, the potential for a bitter taste presents a sensory challenge for peptide-containing products. Due to their amino acid composition, many peptides have a naturally bitter flavor, which can be off-putting to consumers. This bitterness can limit the peptide’s acceptance and marketability, requiring the development of effective masking agents or flavor-modification techniques to enhance the consumer appeal and product success.

Encapsulation methodologies have surfaced as a promising strategy to augment the stability, preserve the functionalities, and modulate the controlled release of bioactive peptides, which hold substantial potential for nutraceutical and food-related applications. By encapsulating peptides within nanocarriers, several advantages can be achieved, including masking their bitter taste to enhance the sensory properties, increasing the solubility, and reducing the hygroscopicity for improved storage preservation [175].

Moreover, bioactive peptides, known for their antimicrobial and antioxidant properties, represent excellent natural food preservatives. Bioactive peptides can be incorporated into packaging materials, such as edible films and coatings, and can further enhance food safety and prolong the shelf life of various food products. This innovative approach offers potential benefits for the food industry by creating safer and more stable food products with enriched functionalities.

## 15. Conclusions

This review has comprehensively examined the multifaceted aspects of fish skin-derived gelatin and gelatin peptides, highlighting their preparation techniques, bioactive profiles, mechanistic functions, and stability enhancement methods. The extensive evaluation of various extraction and processing methods underscores their pivotal role in determining the peptide composition and functional properties of gelatin, which is crucial for optimizing their application in diverse fields. The elucidated bioactivities, including antioxidant, antimicrobial, anti-inflammatory, and anticancer properties, reveal the significant potential of fish skin-derived gelatin peptides as multifunctional ingredients in functional foods, pharmaceuticals, and nutraceuticals.

Mechanistic insights into the interactions of gelatin peptides with biological targets provide a deeper understanding of their bioactive roles, which is essential for harnessing their full therapeutic potential. Furthermore, exploring stability enhancement strategies, such as encapsulation, chemical modification, and advanced delivery systems, offers promising avenues for extending the shelf life and maintaining the bioactivity of gelatin peptides, thereby improving their practical applicability.

In conclusion, fish skin-derived gelatin and gelatin peptides represent a promising and versatile bioresource with substantial potential for innovation and application in food science, health, and biotechnology. This review consolidates current knowledge and identifies critical areas for future research, encouraging further exploration and development of fish skin-derived gelatin peptides to fully realize their benefits in industrial applications.

## Figures and Tables

**Table 1:** Comparative analysis of fish sources, extraction methods, and gelatin yields.

Fish	Extraction Method	Yield (%)	Reference
Black tilapia (*Oreochromis mossambicus*) Red tilapia (*Oreochromis nilotica*)	Pretreatment with NaOH (2%, *w*/*v*), H_2_SO_4_ (2%, *w*/*v*) and citric acid (1%, *w*/*v*); hot water 45 °C for 12 h	5.39 7.81	[36]
Nile perch (*Lates niloticus*)	Pretreated with H_2_SO_4_ (0.01 M); hot water 50, 60 and 70 °C for 5 h	1.2–4.9	[37]
Shortfin scad (*Decapterus macrosomia* Sin croaker (*Johnius dussumieri*)	Pretreatment with NaOH (0.2%, *w*/*v*), H_2_SO_4_ (0.2%, *w*/*v*) and citric acid (1%, *w*/*v*); hot water 40–50 °C for 12 h	7.25 14.3	[38]
Grass carp (*Catenopharyngodon Idella*)	HCl (1.19%, 24 h) pretreatment followed by hot water extraction at 52.61 °C for 5.12 h	19.83	[39]
Farmed giant catfish (*Pangasianodon gigas*)	NaOH (0.2 M)-treated skin soaked in acetic acid (0.05 M) and stirred for 3 h at 25 °C	20.1	[40]
Hoki (*Macruronus novaezelandiae*)	0.75 M NaCl pretreatment for 9 min and hot water extraction at 49.3 °C for 60 min	17.6	[41]
Nile tilapia (*Oreochromis niloticus*)	Alkali-treated (NaOH 3.2% *w*/*v*, 2.3 h) skin soaked in HCl (0.7%, 84 min) followed by hot water extraction 60 °C for 3 h	19.3	[42]
Rainbow trout (*Oncorhynchus mykiss)*	Pretreated with NaOH (0.19 N) and acetic acid (0.121 N) for 3 h, followed by hot water extraction of 50 °C for 16 h	9.36	[43]
Pink perch (*Nemipterus japonicas*) Tiger-toothed croaker (*Otoliths ruber*)	Pretreated with NaOH (0.2%, *w*/*v*), H_2_SO_4_ (0.2%, *w*/*v*), and citric acid (1%, *w*/*v*) for 40 min, followed by hot water extraction at 45 °C for 12 h	5.57 7.56	[44]
Splendid squid (*Loligo formosana*)	Skin soaked in NaOH (0.05 M, 6 h) and phosphoric acid (0.05 M, 24 h) followed by hot water extraction at different temperatures (50, 60, 70, and 80 °C) for 12 h.	8.8–45.3	[45]
Cuttlefish (*Sepia officinalis*)	Alkali-pretreated skin (NaOH, 0.05 M) subjected to 5, 10 and 15 U pepsin/g for 18 h at 4 °C, pH 2	6.59–9.22	[46]
Dog shark (*Scoliodon sorrakowah*) Rohu (*Labeo rohita*) Skipjack tuna (*Katsuwonus pelamis*)	Pretreated with NaOH (0.1 M) and acetic acid (0.2 M) followed by hot water at 45 °C for 12 h	19.7 11.3 17.2	[47]
Farmed amur sturgeon (*Acipenser schrenckii*)	Alkali (NaOH, 0.1 M for 6 h, 4 °C) and acid (acetic acid, 0.05–0.2 M, 3–6 h)-treated skin followed by extraction at 50 °C for 1 h	5.04–24.11	[48]
Octopus (*Octopus vulgaris*)	Alkali (0.05 M NaOH, 1 h at 4 °C)-treated skin soaked in glycine-HCl buffer (10 h at 4 °C) added pepsin (0–15 U) incubated at 40 °C for 4 h.	3.21–7.78	[49]
Kumakuma (*Brachyplatystoma flamentosum*)	Pretreatment with NaCl (0.6 M) followed by NaOH (0.3 M), followed by acetic acid treatment (0.02 M) for min	19.7	[50]

**Table 2:** Gel strength-dependent multifunctionality of gelatin in food production.

Application	Bloom Strength (g)	Concentration and Function
Gelatin desserts and gummy bears	175–275	7–9% as gel formation [59].
Sausages, broths, meat products, and canned meats	175–275	1–5% as an emulsion stabilizer and binding agent
Dairy products	150–250	0.2–1.0% as syneresis stabilizer [59].
Frozen foods	200–250	0.1–0.5% reducing water loss agent [59].
Beverage industry	100–20	0.002–0.015% as clarifying agent [59].

**Table 3:** Comparing the characteristics of gelatin from different sources: fish (warm water and cold water), porcine, bovine, and poultry varieties.

Skin Source	Gel Strength (Bloom Grams)	Gelling Temp (°C)	Melting Temp (°C)	Viscosity (cP)	Water Holding (%)	Fat Binding (%)	Emulsion		Foaming		Reference
							EAI (m^2^/g)	ESI (min)	FE (%)	FS (%)	
Cold-water fish gelatin											
Rohu	124	13.8	18.2	2.50	163	360	NA	NA	17.4	10.5	[47]
Gray triggerfish	168.3	NA	NA	NA	~190	~340%	21.44	42.77	123.04	117.22	[69]
Chum salmon	87.7	NA	NA	NA	NA	NA	~24	~100	~119	~103	[70]
Indian carp	367.66	NA	NA	NA	NA	NA	0.87 *		90	61.8	[71]
Rohu	258.0	NA	NA	NA	NA	NA	0.552 *	ND	92	72.9	
White carp	343.0	NA	NA	NA	NA	NA	0.669	NA	80	77.1	
Warm-water fish gelatin											
Yellowfin tuna	426	18.7	24.3	NA	NA	NA	NA	NA	NA	NA	[58]
Skipjack tuna	177	18.7	24.2	4.37	452	452	NA	NA	19.2	14.4	[47]
Dog shark	206	20.8	25.8	5.6	256	347	NA	NA	21.5	17.6	
Unicorn leatherjacket	149.77	NA	NA	NA	NA	NA	39.17	8.58	116.35	111.11	[72]
Snakehead	311.18	NA	NA	3.40	NA	NA	NA	NA	NA	NA	[73]
Catfish	278.72	NA	NA	5.24	NA	NA	NA	NA	NA	NA	
Pangasius catfish	324.53	NA	NA	3.82	NA	NA	NA	NA	NA	NA	
Red tilapia	487.61	NA	NA	1.73	NA	NA	NA	NA	NA	NA	
Porcine gelatin											
Porcine	295	25.6	36.5	NA	NA	NA	NA	NA	NA	NA	[58]
Bovine gelatin											
Hoki	197	NA	16.6	10.8	NA	NA	NA	NA	NA	NA	[41]
Shortfin scad	177	9.9	23.8	NA	NA	NA	NA	NA	NA	NA	[38]
Bovine	216	23.8	33.8	NA	NA	NA	NA	NA	NA	NA	[58]
Poultry gelatin											
Chicken	270.5	NA	39.83	NA	NA	NA	NA	NA	NA	NA	[74]
Duck	210–260	NA	31.2–33.8	56.9–77.8	NA	NA	NA	NA	NA	NA	[75]
Chicken leg	75–85	NA	NA	7 to 8.9	NA	NA	NA	NA	NA	NA	[76]

Note: * mL oil/mg protein, NA—not analyzed.

**Table 4:** Modification of fish skin gelatin for tailoring the techno-functional properties.

Modification	Fish Skin Source	Tailored Properties	Reference
Sucrose, glucose and fructose addition	Tilapia	Characteristics of the structure, the functions, and the capability of stabilizing the emulsion	[24]
Octenyl succinic anhydride modification	Cold-water fish	Structural, functional, and emulsion stability	[112]
Sodium alginate addition	Cod, pollock, and haddock	Foam and emulsion stabilization	[113]
Spray drying with citric acid, adding pretreatment	Seabass (*L. calcarifer*)	Physico-chemical properties and fishy odor	[114]
NaH_2_PO_4_, MgCl_2_, CaCl_2_, and glycerol addition	Yellowfin tuna	Physicochemical properties	[115]
Basil and citronella essential oils addition	Alaska pollock	Structural, morphological, and thermal properties	[116]
Proteins (chitosan) addition	Baltic cod	Physico-chemical properties	[117]
Chitosan and calcium acetate addition	Catfish (*Pungaseous fungaseous*)	Physico-functional and mechanical properties	[118]
Coenhancers (MgSO_4_, sucrose, and transglutaminase) addition	Tiger-toothed croaker	Gel strength and melting point	[119]
Adjust extraction conditions (alkali and acid treatment, water extraction)	Silver carp	Sensory and instrumental characteristics	[120]
Addition of glycerol and sorbitol	Sole (*Solea* spp.)	Sensory characteristics	[121]

**Table 5:** Peptides with diverse functionalities derived from distinct gelatin sources through the action of various proteases.

Source of Skin Gelatin	Enzyme	Mechanism	Peptide Sequence	Reference
(a) Antioxidant activity				
Silver carp	Alcalase, papain and flavoenzyme	Flavorsome hydrolysate has the highest DPPH-scavenging activity	NA	[151]
Catfish	Protamex	Metal-chelation of 82.33%	NA	[152]
Shark	Alcalase	DPPH scavenging activity (IC 50 27.39 mg/mL)	NA	[153]
Carp	Protamex	DPPH) scavenging activity (38.69%) Ferric-reducing antioxidant power (89.23 µM Trolox/mg sample)	Ala-Tyr dipeptide	[23]
Sole fish	Alcalase	DPPH-scavenging activity	NA	[154]
Oneknife unicornfish	Crude protease from *Bacillus* sp.	DPPH-scavenging activity (63%) Ferric-reducing antioxidant power (25.90 Trolox equivalent (mM mg^−1^)	NA	[155]
Nile tilapia	Ginger protease	DPPH radical (97.21%) lipid peroxidation (48.46%)	Gly-Pro-Y-type X-Hyp-Gly-type	[156]
Giant catfish	Viscera peptidase of gait catfish	DPPH ABTS FRAP Ferrous chelating	NA	[157]
Giant catfish	Visceral alkaline proteases from giant catfish, Izyme AL and trypsin	Ferric-reducing power Ferrous-chelating activity ABTS radical-scavenging activity	NA	[158]
Amur sturgeon	Alcalase or flavoenzyme	Lowered lipid oxidation by monitoring the TBRAS level	NA	[159]
Unicorn leatherjacket	Protease from *Bacillus amyloliquefaciens* and Alcalase	ABTS radical-scavenging activity Ferric-reducing antioxidant power	NA	[160]
Tilapia	Papaya crude extract	ABTS radical-scavenging activity Ferrous-chelating activity Hydrogen peroxide-scavenging activity	NA	[161]
Atlantic mackerel	Pepsin	DPPH ~80% FRAP ~130 μmol Trolox equivalent L^−1^	NA	[162]
Amur sturgeon	Alcalase and flavoenzyme	DPPH IC50-5.38 mg/mL ABTS radical cation- IC50 0.008 mg/mL	Pro–Ala–Gly–Tyr (PAGT)	[163]
Thornback ray	-	DPPH 1980 mg/mL	AVGAT	[164]
Horse mackerel	-	DPPH 72.3% Hydroxyl 51.2%	NHRYDR	[165]
(b) Antihypertensive				
Skate	Subtilisin and actinidin	ACE inhibitory activity	1. GRPGNRGE (IC50 0.74 mg/mL) 2. AKDYEVDAT (IC50 0.52 mg/mL)	[166]
Oneknife unicornfish	Crude protease from *Bacillus* sp.	ACE inhibitory activity	NA	[155]
Nile tilapia	Alcalase	ACE inhibitory activity	GIV, GAP*GF, GFA*GPA, SGNIGFP*GPK, GIPGPIGPP*GRP	[167]
Giant catfish	Viscera peptidase of gait catfish	ACE inhibitory activity	NA	[157]
Atlantic mackerel	Pepsin	ACE inhibitory activity (>70%)	NA	[162]
Pacific cod	Pepsin	ACE inhibitory activity	GASSGMPG (6.9 µM) LAYA (1.45 µM)	[168]
Skate	Alcalase/protease	ACE inhibition with IC_50_ values of 4.22 and 3.09 μM	MVGSAPGVL LGPLGHQ	[169]
Salmon	Trypsin	ACE inhibition with IC_50_ value was 18.7 μM	GLPLNLP	[170]
Alaska pollock	-	ACE inhibition with IC_50_ value was 2.65 mM	GPL	[171]
(c) Antimicrobial activity				
Skate	Subtilisin and actinidin	MIC (mg/mL) 0.52 and 1.75 against *Staphylococcus aureus* and *Klebsiella pneumonia,* respectively MIC (mg/mL) 0.46 and 1.44 against *S. aureus* and *K. pneumonia,* respectively	1. GRPGNRGE 2. AKDYEVDAT	[166]
(d) Antidiabetic activity				
Barbel	Commercial proteases	Dipeptidyl peptidase-IV-inhibiting activity Prolyl endopeptidase-inhibiting activity	ND	[172]

Note: NA—not analyzed; the asterisk (*) is used to show that some proline residues in collagen are hydroxylated, meaning they have an extra hydroxyl group (-OH) added. This modification makes it harder to identify the peptides because it changes their mass and behavior in analysis. By marking these modified proline residues with *, we can easily recognize and account for these differences in the sequence.
